# UV-resistant yeasts isolated from a high-altitude volcanic area on the Atacama Desert as eukaryotic models for astrobiology

**DOI:** 10.1002/mbo3.262

**Published:** 2015-07-04

**Authors:** André A Pulschen, Fabio Rodrigues, Rubens T D Duarte, Gabriel G Araujo, Iara F Santiago, Ivan G Paulino-Lima, Carlos A Rosa, Massuo J Kato, Vivian H Pellizari, Douglas Galante

**Affiliations:** 1 Chemistry Institute, Universidade de São PauloSão Paulo, Brazil; 2 Microbiology, Immunology and Parasitology Department, Universidade Federal de Santa CatarinaFlorianópolis, Brazil; 3 Interunities Graduate Program in Biotechnology, Universidade de São PauloSão Paulo, Brazil; 4 Brazilian Synchrotron Light LaboratoryCampinas, Brazil; 5 Department of Microbiology, Universidade Federal de Minas GeraisBelo Horizonte, Brazil; 6 NASA Postdoctoral Program Fellow at NASA Ames Research CenterMoffett Field, California; 7 Oceanographic Institute, Universidade de São PauloSão Paulo, Brazil

**Keywords:** Astrobiology, Atacama Desert, eukaryote, extremophiles, UV radiation, yeast

## Abstract

The Sairecabur volcano (5971 m), in the Atacama Desert, is a high-altitude extreme environment with high daily temperature variations, acidic soils, intense UV radiation, and low availability of water. Four different species of yeasts were isolated from this region using oligotrophic media, identified and characterized for their tolerance to extreme conditions. rRNA sequencing revealed high identity (>98%) to *Cryptococcus friedmannii*, *Exophiala* sp., *Holtermanniella watticus*, and *Rhodosporidium toruloides*. To our knowledge, this is the first report of these yeasts in the Atacama Desert. All isolates showed high resistance to UV-C, UV-B and environmental-UV radiation, capacity to grow at moderate saline media (0.75–2.25 mol/L NaCl) and at moderate to cold temperatures, being *C. friedmannii* and *H. watticus* able to grow in temperatures down to −6.5°C. The presence of pigments, analyzed by Raman spectroscopy, correlated with UV resistance in some cases, but there is evidence that, on the natural environment, other molecular mechanisms may be as important as pigmentation, which has implications for the search of spectroscopic biosignatures on planetary surfaces. Due to the extreme tolerances of the isolated yeasts, these organisms represent interesting eukaryotic models for astrobiological purposes.

## Introduction

The Atacama Desert (Chile) is an extreme environment on Earth, classified as a hyper arid desert, with high UV radiation incidence, scarce sources of organic carbon, large daily temperature variations and low water availability (Navarro-Gonzalez et al. 2003). As an example, de los Rios and collaborators registered a variation in the temperature from 46.5°C to −8.00°C, during 1 year, and a minimum of humidity of 1.40% at the Salar de Yungay (De los Ríos et al. 2010). These harsh conditions make the Atacama Desert a challenging place for life and a good analog of extraterrestrial environments, such as Mars (Navarro-Gonzalez et al. 2003; Cabrol et al. 2007). The Sairecabur volcano, located on the San Pedro de Atacama region, near the Salar de Atacama, is an example of such distinct regions, being oligotrophic, arid, with high daily temperature variations and occurrence of only occasional snowfalls. The absence of permanent snow cover or glaciers is indicative of the exceptionally dry climate of such high-altitude regions (Costello et al. 2009; Lynch et al. 2012). In addition, the incidence of Solar radiation is higher at greater altitudes (Cabrol et al. 2014), including deleterious UV radiation, thus producing an environment restrictive to most living organisms (Lynch et al. 2012). Indeed, several works concerning the microbiology of high altitudes focus on the effect of UV radiation (Zenoff et al. 2006; Libkind et al. 2009; Ordonez et al. 2009). In addition, microorganisms living at high altitude must also deal with low temperatures, around the freezing point of water, and desiccation stress, due to the low atmospheric pressure and humidity.

The microbial diversity of the Atacama Desert has been studied over the last years, focusing mainly on the bacterial (Drees et al. 2006; Connon et al. 2007; Okoro et al. 2009; Neilson et al. 2012) and cyanobacterial diversities (Warren-Rhodes et al. 2006; Wierzchos et al. 2006; Azua-Bustos et al. 2012). Prokaryotes have been studied regarding their tolerances to several stressors present in the Atacama, including the inactivation by environmental-UV (Dose et al. 2001; Cockell et al. 2008), growth at several concentrations of salt (Rivadeneyra et al. 1999; Cockell et al. 2010) and desiccation (Billi 2009). In addition, some isolates from the Atacama were shown to be resistant to some stressors that are not found on the terrestrial environment, such as UV-C and ionizing radiation (Billi et al. 2000; Paulino-Lima et al. 2013). The resistance of those organisms to extreme conditions makes them interesting candidates for astrobiological studies, as model-organisms to survive on harsh extraterrestrial environments. For this purpose, the high-altitude regions in the Atacama have a strong potential to be further explored (Cabrol et al. 2009).

However, even considering all the efforts in characterizing the microbial diversity of the Atacama, few works have investigated the presence and adaptive mechanisms of eukaryotes at the desert (Conley et al. 2006). Therefore, the goal of this work was to isolate and characterize yeast strains from soil samples collected at the Sairecabur volcano, and to submit the isolates to different laboratory-simulated extreme conditions. More specifically, the strains were exposed to different fluences of environmental-UV, UV-B and UV-C radiation; photoprotective pigments were characterized by Raman spectroscopy and analyzed for their role on the UV resistance, as well as the capability of the strains to grow in moderate to high salt concentrations and at different temperatures. The results demonstrate the adaptive competence of such organisms to conditions found in the Atacama Desert, and even harsher ones, which could be present on extraterrestrial environments, contributing to expand our knowledge of the adaptations of eukaryotes in extreme conditions, and proposing new models to be used in astrobiological studies.

## Material and Methods

### Site and soil characterization

**Figure 1:**
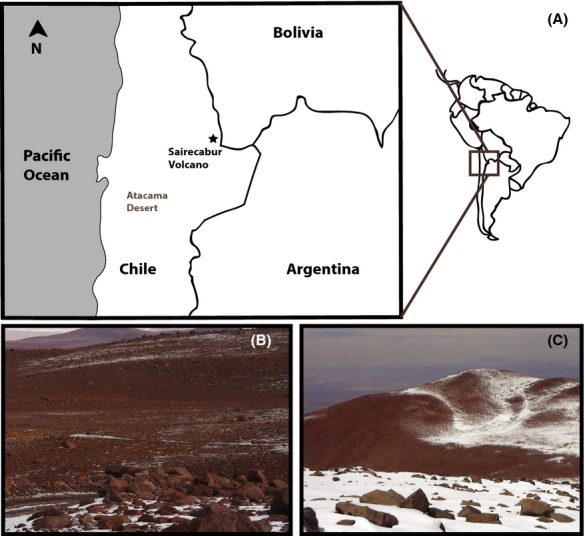
Location of Sairecabur volcano, in the Atacama Desert, Chile (A). At the time of the sample collection, temperatures were below the freezing point and some areas of the volcano were covered with snow (B and C).

Samples were collected from the top layer of soil at three different sites of the Sairecabur volcano, Atacama Desert (Fig.1), in January 2012, using sterile tools, placed in sterile 50 mL tubes, sealed and kept refrigerated until the analysis. The three soil samples used in this work were collected in the following sites (latitude, longitude and altitude, respectively): *S5047* (soil from volcano slope) = 22.716945°S/67.923690°W/5047 m; *S3981* (soil from volcano slope) = 22.706917°S/67.996050°W/3981 m; *S4823* (sulfur-rich soil) = 22.715898°S/67.933632°W/4823 m.

Measurements of UV-A and UV-B fluxes at the volcano were made at 5091 m using a portable radiometer (Vilber Lourmat VLX-3W, Marne-la-Vallée, France). For comparison, additional measurements were made with the Sun on the zenith, on the same season, at the Salar Yungay (Atacama, Chile – 24.115012°S/69.880035°W/Altitude: 948 m) and in a region of similar latitude but of sub-tropical climate, in Brazil (São Paulo state, Brazil – 23.00464°S/46.964807°W/850 m).

The collected samples were characterized by measuring the pH using the methodology described by Barrett et al. (2004). Salinity was evaluated by measuring the conductivity, as described by Okoro et al. (2009). X-ray fluorescence (XRF) was used to determine the elemental composition of the soil samples (Table1). The XRF measurements were made at the XRF beamline of the Brazilian Synchrotron Light Laboratory (LNLS) (Perez et al. 1999) in the microbeam mode, using polychromatic excitation and an elliptical capillary for focusing the beam on a spot of about 50 *μ*m diameter, with the final spectra being averages of at least five different points to minimize intrinsic inhomogeneity. All data were treated using the PyMCA software (Sole et al. 2007) for the calculation of the absolute values of concentration for each element.

**Table 1:** pH, conductivity and elementary composition (in mass concentration, obtained with X-ray fluorescence, for *Z* ≥ 15) of the soil samples

	1. Slopesoil 5047 m (S5047)	2. Slopesoil 3981 m S3981)	3. Sulfur-rich soil 4823 m (S4823)
pH	4.25	5.36	3.34
Conductivity	11.6 *μ*S/cm	83.9 *μ*S/cm	376 *μ*S/cm
S	–	–	15.8%
Cl	–	–	–
K	0.41%	0.02%	5.32%
Mn	0.02%	–	0.05%
Fe	1.07%	0.15%	3.48%
Cu	–	–	–

Trace elements (–) are here defined as those with concentrations lower than 0.01%.

### Isolation and molecular identification

Yeast colonies were isolated using a mineral, organic-poor medium, composed of (NH_4_)_2_SO_4_, 0.4 g L^−1^; KH_2_PO_4_, 0.5 g L^−1^; CaCl_2_, 0.25 g L^−1^; MgSO_4_, 0.5 g L^−1^; Na_2_S_2_O_3_, 5 g L^−1^, and FeSO_4_, 0.01 g L^−1^; 15 g L^−1^ DifcoBacto Agar; pH adjusted to 4.8 (all reactants were purchased from Synth (Diadema, São Paulo, Brazil)). To extract yeast cells from the soil, 2 g of the sample was transferred to flasks with 20 mL of sterile 0.9% w/v (roughly 0.15 mol/L) NaCl saline solution (pH 4.8) and shaken at 150 rpm for 24 h at 30°C or 4°C. Aliquots of 100 *μ*L were spread on mineral medium and incubated at 30°C and 4°C. Yeast colonies were then transferred to TGY plates (yeast extract, 5 g L^−1^; tryptone, 5 g L^−1^ and glucose, 1 g L^−1^) with pH adjusted to 4.8.

Yeast identification was carried out by sequencing the D1–D2 variable domains of the large rRNA subunit gene using the primers NL1 (5′ GCATATCAATAAGCGGAGGAAAAG) and NL4 (5′ GGTCCGTGTTTCAAGACGG), as described by Rosa et al. (1999) and were confirmed by the amplification of the internal transcribed spacer (ITS) and ITS4. After amplification, the purified PCR fragments were sequenced with an ABI 3130 Genetic Analyzer automated sequencing system (Life Technologies, Carlsbad, CA). The obtained sequences were analyzed using the GenBank database and the nearest species found were used in the molecular analysis (Table2). Yeasts were stored on GYMP broth (glucose, 20 g L^−1^; yeast extract, 5 g L^−1^; malt extract, 5 g L^−1^; Na_2_PO_4_, 2 g L^−1^) with 20% of glycerol at −80°C and deposited in the Culture Collection of Microorganisms and Cells at the Universidade Federal de Minas Gerais – UFMG.

**Table 2:** Growth range, halotolerance, and molecular identification of the isolated yeasts used on the resistance experiments, using BLASTn and ITS region

Isolate ID	Sample ID	Primer	Result top BLAST (access no. GenBank)	Identity (%)	No. of bp analyzed	Species or proposed taxonomic group (GenBank access no.)	Halotolerance (NaCl)	Max. temperature with observable growth	Min. temperature with observable growth
16Lv2	S5047	NL	*Cryptococcus friedmannii* (JX092255)	100	599	*Cryptococcus friedmannii* (KM243310)	1.75 mol/L	25°C (weak)	−6.5°C
		ITS	*Cryptococcus friedmannii* (AF145322)	99	549	*Cryptococcu sfriedmannii* (KM243311)			
15Lv1	S4823	NL	*Exophiala capensis* (JF499861)	99	587	*Exophiala* sp. (KM243304)	1.25 mol/L	25°C	0°C
		ITS	*Exophiala* sp. (HQ452332)	99	509	*Exophiala* sp. (KM243305)			
16Lv1	S3981	NL	*Holtermanniella watticus* (KC006859)	99	601	*Holtermanniella watticus* (KM243308)	2.25 mol/L	25°C (weak)	−6.5°C
		ITS	*Holtermanniella watticus* (JQ857031)	99	501	*Holtermanniella watticus* (KM243309)			
16Lv3	S3981	NL	*Rhodosporidium toruloides* (GQ169736)	99	587	*Rhodosporidium toruloides* (KM243312)	0.75 mol/L	30°C	10°C
		ITS	*Rhodosporidium toruloides* (JN246549)	98	524	*Rhodosporidium toruloides* (KM243313)			

ITS, internal transcribed spacer.

1 Coverage of 100% for all sequences analyzed.

### Raman spectroscopy and characterization of pigments

To investigate the presence of photoprotective pigments, mainly melanin and carotenoids, the isolates were analyzed by Raman spectroscopy, using a Renishaw (Renishaw PLC, Wotton-under-Edge, UK) inVia micro-Raman Spectrometer with HeNe laser line (633 nm), 20× objective and CCD detector, and a Bruker (Bremen, Germany) RFS 100/S FT-Raman with incident radiation of 1064 nm and a LN_2_-cooled Ge detector. The samples were analyzed without further preparation, by removing the colonies from the plates and depositing them on glass slides. Low laser power was used to avoid thermal or photochemical damage.

### UV-C and UV-B resistance experiments

The UV-C resistance of the yeasts was evaluated in comparison to *Saccharomyces cerevisiae*, strainBY4742, used as low-radiation resistant model. Since there is no consensual UV-resistant model for yeasts, it was used the radioresistant bacterium *Deinococcus radiodurans* (R1 type strain, obtained from Instituto de Radioproteção e Dosimetria – IRD, Rio de Janeiro, Brazil) to validate the experiment. The yeasts were grown in liquid TGY medium, pH 4.8, under 150 rpm shaking, at 10°C for *Exophiala* sp. 15Lv1, *Cryptococcus friedmannii*, and *Holtermanniella watticus*, at 30°C for *S. cerevisiae* and *Rhodosporidium toruloides*, and at 30°C and no pH adjustment for *D. radiodurans*.

Cells were grown to OD_595_ of about 0.6–0.8, washed twice with 0.9% w/v NaCl solution (pH corrected to 4.5 for the yeasts, and neutral for *D. radiodurans*). The pellet was dispersed in sterile saline solution and adjusted to a final concentration of 10^6^–10^7^ cells/mL. A volume of 10 mL of this cell suspension was transferred to a 10 cm diameter sterile Petri dish and irradiated under orbital shaking with a Philips (Philips, Eindhoven, The Netherlands) TUV-20W low-pressure Hg lamp (253.7 nm). The irradiation was monitored during the experiment using a calibrated radiometer (Vilber Lourmat RMX-3W) and a UV-C photocell (CX-254, Vilber Lourmat). The UV-C flux measured during the irradiation was 6.0 W/m² and the sample was placed at 25 cm from the lamp. No substantial temperature variation that could affect cell survival was detected in the solution during the experiment. After the different fluences, the colony-forming units (CFU) were evaluated by incubation on TGY agar plates at 10°C or 30°C in the dark. All experiments were made in triplicates.

The UV-B experiment was carried out in a similar way, using the same radiometer with an UV-B photocell (CX-312, Vilber Lourmat) to monitor the fluence. Two LightTech Narrow Band UV-B 20 W Hg lamps and one Philips TL20W Hg lamp with major line-emission at 312 nm were used to generate the UV-B radiation, with a measured intensity of 16.5 W/m^2^. Since more time under UV-B radiation was needed to generate enough biological damage, 7 cm diameter Petri dishes, surrounded by wet cotton, were used to counterbalance the evaporation rate. On each plate, 6 mL of cell suspension were added, and no variation on the temperature of the solution was observed during the exposure. The survival was evaluated by CFU counting, and the experiments were performed in triplicates. The lamps, as measured with the UV-C photocell, emitted no UV-C.

### Environmental-UV radiation tolerance experiment

The environmental-UV experiment was designed to approximate the conditions found on the natural environment on Earth. The exposures were performed directly over agar, since the microorganisms were collected from solid substrate, not from a solution. In addition, in natural environments (specially the more restrictive ones), microorganisms, including yeasts, are likely to exist in a low metabolic, quiescent state, more similar to the stationary phase (Gray et al. 2004; Navarro Llorens et al. 2010). Previous authors, working with high-altitude environments and performing UV experiments have also preferred not to irradiate the cells during active growth (Zenoff et al. 2006). With that in mind, the cells were grown in TGY media to the beginning of the stationary phase. For *C. friedmannii, H. watticus, R. toruloides* and *S. cerevisiae,* OD_595_ ∼ 0.480 (after 1:20 dilution), for *Exophiala* sp.15Lv1, OD_595_ ∼ 0.900 (after 1:20 dilution) and for *D. radiodurans* OD_595_ ∼ 0.670 (after 1:10 dilution). The suspension of washed cells was spread on TGY pH 4.8 agar plates (or TGY pH 7 for *D. radiodurans*) at different dilutions (10^7^–10^3^ cells/mL) and the plates were exposed (without lid) to simulated environmental-UV radiation, using an Oriel® (California, USA) Sol UV-2 Solar simulator (85.7% UV-A, 11% UV-B and 3.3% of visible light). The flux during the irradiation was 96.0 W/m^2^ for UV-B and 131.5 W/m^2^ for UV-A measured with a Vilber Lourmat radiometer with UV-B and UV-A photocells (CX-312 and CX-365, Vilber Lourmat). The plates were exposed for 10, 20, 30, and 40 min, at room temperature (∼20°C) and then incubated. The survival was evaluated by CFU counting, in triplicates. Once again, using the UV-C photocell, we certified that the simulator emitted no significant UV-C during the irradiation procedure (see Fig. S3 for the complete spectrum).

### Temperature growth range, halotolerance, and oligotrophy

The temperature experiment was performed with cultures on TGY pH 4.8 agar plates incubated at 0, 4, 10, 15, 20, 25, and 30°C and in TGY pH 4.8 liquid medium under agitation with anti-freezing solutes for temperatures ranging from 0 to −6.5°C (Chin et al. 2010). The growth curves were recorded at 0°C and −3°C with the flasks initially inoculated to OD_595_ = 0.2 from a previous growth performed at 0°C. For the growth curve at −6.5°C, flasks were inoculated also to an initial OD_595_ = 0.2 from a previous growth at −3°C with glycerol 0.4 mol/L (w/v). The high initial optical density was used to allow a faster evaluation of the yeasts’ development, since flasks with small initial optical densities would take longer times to show evidences of growth of the organism, especially at low temperatures. To minimize freezing, the experiments at −3°C, were performed using either 0.5 mol/L of NaCl (w/v), a kosmotropic solute, or 0.4 mol/L of glycerol (w/v), a chaotropic solute (Chin et al. 2010). At −6.5°C, it was used 0.6 mol/L (w/v) of glycerol.

For the studies concerning the halotolerance, agar plates of saline TGY medium pH 4.8 were prepared with different NaCl concentrations: 0.50, 0.75, 1.00, 1.25, 1.50, 1.75, 2.00, 2.25, and 2.50 mol/L. To ensure proper solidification, it was used 24 g L^−1^ of agar. The inoculum on the saline medium was made from recently cultured nonsaline plates (TGY medium), and CFU was evaluated. Finally, since the yeasts were isolated in culture media without the addition of any carbon source, the growth capacity in low nutrient conditions of the isolates was confirmed with a similar methodology to the one used by Uetake et al. (2012), using ultra-pure water agar medium (UWA). The medium was prepared with 1.5% w/v of Difco Bacto Agar and ultrapure water (Milli-Q®, from Millipore (Molsheim, France) Direct-Q system), with pH adjusted to 4.8 with a 0.5 mol/L H_2_SO_4_ solution. The cells were spread on the UWA plates, incubated and the CFU (and the size of the colonies) was evaluated after 20 days.

## Results and Discussion

### Soil samples and environmental conditions

Some of the physicochemical characteristics of the sampling sites are shown in Table1. The data obtained from XRF corroborates the observation that S4823 consists in a sulfur-rich soil. In addition, this was the most acidic sample studied, with the low pH of volcanic soils being defined by many factors, as age, precipitation, presence of organic matter, volcanic, and biological activity (Flierman and Brock 1972; Ugolini and Dahlgren 2002; Dahlgren et al. 2004). Since yeasts can grow in acidic media, this might represent an advantage to those organisms at this particular environment.

Concerning the conductivity, Drees et al. (2006), reported a large variation in conductivity values in soils of the Atacama, with lower ones of about 10 *μ*S/cm and higher ones greater than 2000 *μ*S/cm. In the same way, the values found by Okoro et al. (2009) vary from 126 to 1540 *μ*S/cm, being the last one from a salt-rich soil collected at the Valle de la Luna site. In our work, the values found in S5047 and S3981 are comparable with the lower values, while that for S4823 is an average value. The low conductivity obtained in S5047 and S3981 are in accordance with the elemental composition measured, since it was observed a low content of ion-forming species such as chloride, potassium, calcium and sulfur. In S4823 that had a greater value of conductivity, a higher concentration of potassium (5.32%) was measured. In addition, the presence of sulfur-oxidizing microorganisms could be responsible for the increase in conductivity, due to sulfate production, which is coherent with the lower pH, although this was not evaluated on the present work.

It has to be considered, however, that the macroscopic conductivity values and mineral composition may not be exactly the ones existing on the aqueous milieu where microorganisms can be thriving. Although microniches can be present and macroscopic measurements cannot properly analyze these small environments, this data is still useful for a general characterization of the sample.

Considering the environmental radiation measurements, the intensity of UV-B and UV-A increases with the altitude and absence of clouds or water vapor (Blumthaler et al. 1997), hence the dry conditions of the desert and the altitude of the volcano contribute for the intense UV flux. The value measured at 5091 m at the time of the sampling (at noon) was 36.4 W/m^2^ for UV-A and 15.6 W/m^2^ for UV-B. For comparison, the radiation values measured at Salar Yungay (on the hyper arid region of the Atacama, at 948 m) were 29.5 W/m^2^ for UV-A and 11.6 W/m^2^ for UV-B; in Brazil (São Paulo), under subtropical conditions, the measurements were 26.4 W/m^2^ for UV-A and 9.3 W/m^2^ for UV-B radiation.

### Molecular identification, growth and pigment characterization of the isolates

Four different species were identified from the isolated yeasts (Table2). To our knowledge, this is the first report of those species at the Atacama Desert. The yeast *C. friedmannii* and *H. watticus* were both first isolated from the Antarctica samples (Vishniac 1985; Guffogg et al. 2004), and also found in other cold environments, as Iceland and Russia (Vishniac 2006). It is known that the genus *Cryptococcus* is commonly found and adapted to live in deserts (Vishniac 1998), being tolerant to UV, desiccation and nutrient-poor conditions, with some species already described on the Atacama Desert (Vishniac 2006). The yeast *R. toruloides* has been suggested to be adapted to some extreme environments, with low pH and high concentrations of toxic metals (Gadanho et al. 2006), but this is the first time that it is reported in a cold, high-altitude environment. A black yeast species was isolated from S4823, however, the analysis of the NL and ITS could only state that it belongs to *Exophiala* genus, being other primers at different regions necessary for the identification at the species level. More experiments are being performed by the group and will be presented in a future work. Although some of the same yeast species were isolated from different samples of the volcano (*R. toruloides* in S5047, (Genbank KM243302 for NL and KM243303 for ITS), *C. friedmannii* in S3981, (Genbank KM243300 for NL andKM243301 for ITS) and also in S4823 (KM243306 for NL andKM243307 for ITS), the full set of resistance experiments was performed only with one strain of each species (described on Table2). Preliminary experiments demonstrated that there were no major differences on the biological responses between the same species isolated from different samples.

The qualitative results concerning halotolerance, oligotrophic growth, and growth temperatures are presented in Table2. Although the volcanic soils presented low to moderate salinity in a macroscopic scale, the salt tolerance of the isolates was evaluated to assess their potential for astrobiological studies, since salt deposits were discovered on Mars (Osterloo et al. 2008). Concentrations of 0.6 mol/L NaCl are already toxic for *S. cerevisiae*, for example (Prista et al. 1997). *Rhodosporidium toruloides* was shown to possess the lowest NaCl tolerance among the yeasts isolated from the volcano, being 0.75 mol/L of NaCl already enough to diminish greatly the formation of colonies. The other yeasts have shown greater tolerance, being *H. watticus* the most salt-tolerant one, capable of growth even at 2.25 mol/L of NaCl. According to Vishniac (1998), there is no obvious correlation between yeasts found in arid soils and deserts and osmotolerance, since the low organic content in these soils is not normally compatible with the high energy requirements for halotolerance in yeasts. Low growth rates have also been shown to have an anti-correlation with halotolerance.

Uetake et al. (2012) have recently reported the isolation of oligotrophic yeasts from the Gulkana glacier (Alaska, USA) using UWA media, suggesting that those yeasts were capable of oligotrophic growth on sites where liquid water was present on the glacier. Using the same organic-poor medium, *H. watticus, C. friedimannii*, and *Exophiala* sp.15Lv1 showed the capacity to generate 1 mm colonies after 20 days. The yeast *R. toruloides* was also capable of developing colonies at UWA media, but they were smaller when compared with the other yeasts, no bigger than 0.5 mm. The capacity of development at low nutrient conditions is interesting for astrobiological purposes, but it can also represent an advantage for prevailing at organic poor soils, which constitutes one of the limiting factors for life at volcanic areas at the Atacama Desert (Lynch et al. 2012).

Three of the yeasts have shown growth at low temperatures (shown in Fig. S1), including at 0°C. However, only *H. watticus* and *C. friedmannii* have shown growth at −3°C and −6.5°C. It was also observed a slow growth of *Exophiala* sp.15Lv1 at 0°C, but not at lower temperatures. Using NaCl, a kosmotropic solute (which disfavors growth of fungi at lower temperatures [see Chin et al. 2010]), it was observed a diminishment of the optical density for longer periods of incubation. Using the chaotropic solute glycerol, there was no increase at the optical density after 14 days, but after this period, the *Exophiala* flasks started to freeze (for unknown reasons). As it was possible to have good growth with the tested solutes, on these concentration, at the optimum temperature for the yeasts (although with a small delay for NaCl, see Fig. S1), the observed effects at the experiment were mostly due to the temperature, discarding interferences from the anti-freezing agents. To confirm the capability of *Exophiala* sp.15Lv1 to grow at temperatures below 0°C, higher concentrations of glycerol and longer incubations periods might be needed, due to the slow growth behavior of this isolate. Although the *R. toruloides* strains isolated from the volcano did not develop at 4°C and 0°C, and it was observed that colony growth at 10°C induced a change on the pigmentation from orange to an intense pink, which might indicate a stress-response strategy of *R. toruloides* to the high altitude and cold environment of the volcano.

The Atacama Desert presents great daily temperature variations, but due to the high altitude, the temperatures are mainly colder than in lower areas, being the capacity to develop at temperatures as low as 0°C a possible advantage to such organisms. In addition, as has been already pointed out by Chin et al. (2010), the presence of chaotropic salts found on Mars may favor the survival of cold, tolerant microorganisms at lower temperatures. Recently, Fischer et al. (2014), studying the chaotropic salts NaClO_4_ and Ca(ClO_4_)_2_ have demonstrated that regions where salts and ice coexist in Mars might form liquid brines temporarily, which could allow microbial growth to thrive, even on the surface of that planet. Also, according to Osterloo et al. (2008), chlorides salts are globally spread on Mars. Thus, the capability of thriving at temperatures below the freezing point in chaotropic solutes (as glycerol) or in kosmotropic solutes (as NaCl) as observed by *C. fredmannii* and *H. watticus,* as well the salt tolerance of the isolates, are also important when considering these yeasts by an astrobiological perspective.

**Figure 2:**
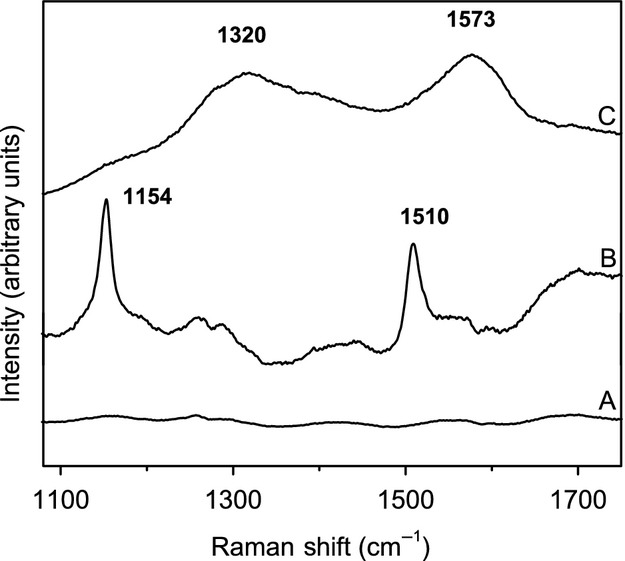
Raman spectra of the yeast cultures: (A) *Cryptococcus friedmannii*. Similar spectra with no evident peaks were acquired for *Holtermanniella watticus* and *Saccharomyces cerevisiae*, not shown here; (B) *Rhodosporidium toruloides*. Similar spectra were acquired for *Deinococcus radiodurans* and therefore not shown; (C) *Exophiala* sp. 15Lv1.

In order to further characterize each isolate, pigment analysis using Raman spectroscopy was performed. This technique is being used in the literature in the search of biosignatures in the context of astrobiology (Ellery and Wynn-Williams 2003; Edwards et al. 2005) and it can be a simple analytical method to analyze the presence of some categories of photoprotective pigments with minor preparation of the samples. In the present work, the spectra (Fig.2) were obtained directly from colonies growing on solid culture media, without further processing.

The two strong peaks at *ca*. 1150 and 1510 cm^−1^ in the Raman spectra, as observed in Figure2B, are well described on the literature as corresponding to carotenoids, more specifically to the C=C–C stretch modes (De Oliveira et al. 2010). Since carotenoids are widespread in different living systems, including several kinds of microorganisms, and their Raman bands are very intense, they are being proposed as one potential spectroscopic biosignature for the detection of extant life on Mars (Parnell et al. 2007). The two broad bands at *ca*. 1320 and 1570 cm^−1^, as observed in Figure2C, can be attributed to the class of melanin (Perna et al. 2013). The laser power was kept low to avoid damaging the samples, and a threshold test was performed to ensure that the 1300/1600 bands are not caused by the presence of D and G carbon bands normally observed in the decomposition of organic matter. The failure to find well-defined bands on the spectra of the other samples, as on Figure2A, should not be interpreted as the total absence of pigments, since they can be present in minor quantities, below the detection limit of this technique in the conditions of the measurement (Jehlicka et al. 2014). In all the cases, the interpretation of the Raman spectra is in agreement with the color of the colonies grown on TGY media: orange for *R. toruloides*, black to *Exophiala* sp. and pale-beige for *C. friedmannii* and for *H. watticus*.

Carotenoids and melanins are known as UV protective pigments (Singaravelan et al. 2008; Libkind et al. 2009) since they are capable of absorbing UV radiation at different wavelengths, including on the UV-C (Wynn-Williams and Edwards 2002). In addition, carotenoids can scavenge free radicals generated by UV light, protecting the cell against oxidative damage (Moore et al. 1989; Schroeder and Johnson 1993). It is important to state, however, that other pigments or photoprotective compounds without intense Raman bands can also be present in the samples, without being detected by this technique, due to characteristics of the samples (such as strong fluorescence).

### UV exposure experiments and survival profile of the isolates

Any microorganism that could inhabit the Martian surface would be exposed to intense ultraviolet radiation from the Sun, including UV-C radiation, due to the lack of an ozone layer (Paulino-Lima et al. 2013), unless it was shielded by soil or dust. UV-C radiation (200–280 nm) was probably present on the environment of the Archaean Earth, before the rise of oxygen in the atmosphere (Cockell 1998). It is still abundant above the ozone layer in the present day Earth’s stratosphere, where some microorganisms have been detected since 1936 (Smith 2013). UV-C radiation is very effective to inactivate microorganisms, being commonly used for sterilizing surfaces, as used on most of the germicidal lamps. Therefore, it is an easily accessible tool to select extremely radiation-resistant microorganisms. UV-B radiation (280–320 nm) is the most biologically active form of UV radiation reaching the surface of present-day Earth (Björn 2008), however, it is easily scattered by clouds in the atmosphere. Since UV-A radiation (320–400 nm) is the most abundant of the Solar UV spectrum, it is usually considered the most important for practical purposes on natural environments (Björn 2008).

Both UV-C and UV-B portions of the electromagnetic spectrum are capable of generating cyclobutane pyrimidine dimers (CPDs) and (6-4) pyrimidine-pyrimidine (Douki et al. 1997), which can lead to DNA breaks due to inefficient repair (Santos et al. 2013). UV-C radiation is more efficient than UV-B in generating DNA photoproducts (Mitchell et al. 1991; Ravanat et al. 2001). However, UV-B has a higher efficiency when compared to UV-C radiation in generating reactive oxygen species (ROS) (Santos et al. 2013), which leads to cell death by damaging not only DNA, but also other cell components, as lipids and proteins (Daly 2012; Santos et al. 2013).

Although UV-A radiation can also induce DNA photoproducts, like CPDs, it is less efficient than UV-C and UV-B (Cadet et al. 2012) with its lethal biological effects occurring mainly due to generation of ROS species (Hoerter et al. 2005; Santos et al. 2013). An important oxidative base modification generated under UV-A irradiation, 7,8-dihydro-8-oxoguanine (8-oxoG), occurs through oxidative damage of the DNA and has great impact on mutagenesis and cell survival of *S. cerevisiae*, for example. In this scenario, CPDs have minor importance when compared to 8-oxoG (Kozmin et al. 2005), although experiments with several mammalian cells suggests that CPDs plays important roles in UV-A genotoxicity in such models (Cadet et al. 2012). Also, the combined exposure of UV-A and UV-B radiation can lead to the photoisomerization of 6,4-PPs into Dewar valence isomers, a third class bipyrimidine photoproduct, and important when considering microbial survival under environmental UV exposure (Meador et al. 2014).

**Figure 3:**
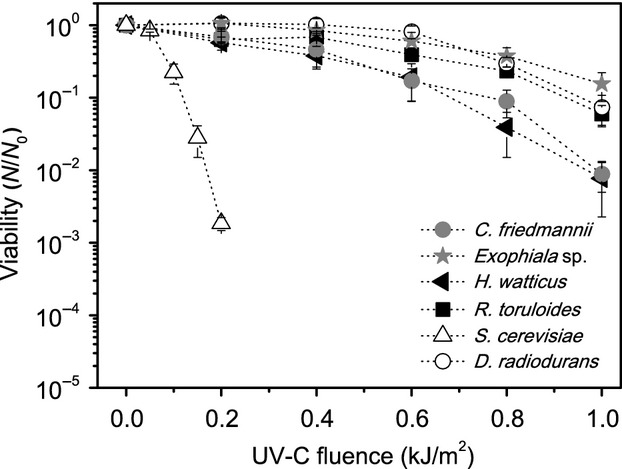
Survival curves for UV-C radiation of the tested organisms. The error bars indicate the variance between the triplicates.

**Figure 4:**
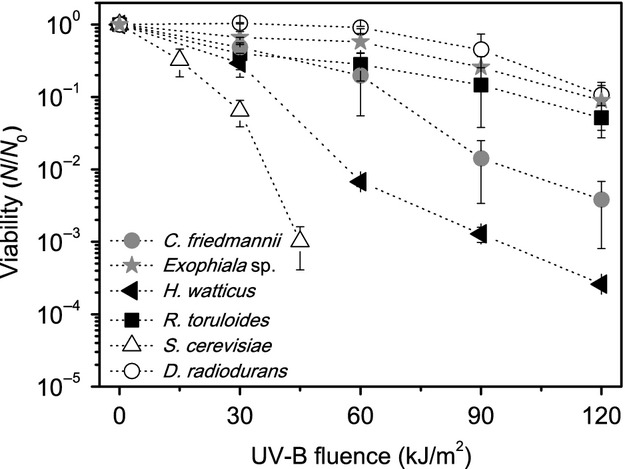
Survival curves for UV-B radiation of the tested organisms. The error bars indicate the variance between the triplicates.

In our experiments, it was observed that all isolates were highly resistant to UV-C and UV-B, especially when compared to *S. cerevisiae*, being some of the survival profiles similar to *D. radiodurans* (Figs.3, 4).

The survival of the black yeast *Exophiala* sp.15Lv1 and *R. toruloides* was higher than the negative control *S. cerevisiae,* even matching that of *D. radiodurans*. Using our experimental protocol, *Exophiala* sp. and *R. toruloides* only lost one log of viability (measured by CFU counting) after exposure to 1 kJ/m^2^, while *S. cerevisiae* lost viability with smaller fluences (almost three logs drop of CFU counts after 0.2 kJ/m^2^ of exposure). The yeasts *C. friedmannii* and *H. watticus* also scored greater survival, losing 90% of viability only after 0.6 kJ/m^2^. Regarding the UV-B radiation, *Exophiala* sp.15Lv1 and *R. toruloides* have also shown similar resistances to that of *D. radiodurans* and greater survival when compared to *S. cerevisiae*. However, the difference of survivability in this wavelength range among the isolated yeasts and the negative control (*S. cerevisiae*) appears to be smaller than on the UV-C. These differences might reflect the biological effects caused by UV-C and UV-B radiation.

Since the intensity and effects at the cell level are different, the yeast cells must display different mechanisms to deal or avoid those damages, being one of them the presence of photoprotective pigments. In our experiments, the two pigmented yeasts (*Exophiala* sp.15Lv1, melanin rich and *R. toruloides,* carotenoid rich) have shown the greatest survival under UV-B and UV-C irradiation, when compared to the yeasts without detectable bands attributed to pigments on the Raman spectra (*C. friedmannii* and *H. watticus*). Moline et al. (2009), comparing pigmented and natural albino strains of yeasts, have proposed that carotenoids enhanced the UV-B resistance on those organisms. Regarding melanin, Wang and Casadevall (1994) have found that the presence this pigment has enhanced the UV-C resistance of melanized *Cryptococcus neoformans,* when compared with the nonmelanized cells. However, Schiave et al. (2009) reported that there is small or no difference between melanized and nonmelanized cells of *C. neoformans* and *Cryptococcus laurenti* after UV-B exposure, questioning the capacity of melanin to provide protection against UV-B.

Although the pigmented yeasts tested in this work were more resistant to UV-C and UV-B than the nonpigmented ones, the presence of carotenoids and melanin itself cannot ensure the survival against radiation, as it does not completely block UV from affecting the DNA (Sinha and Hader 2002). Thus, complementary cellular mechanisms should be present. This is corroborated by our data, as on the survival curves of *H. watticus* and *C. friedmannii* (Figs.3, 4), which, despite the lack of signal of the bands attributed to pigments on Raman spectra in our growth conditions, still presented a significant resistance to UV-B and, especially, to UV-C radiation.

**Figure 5:**
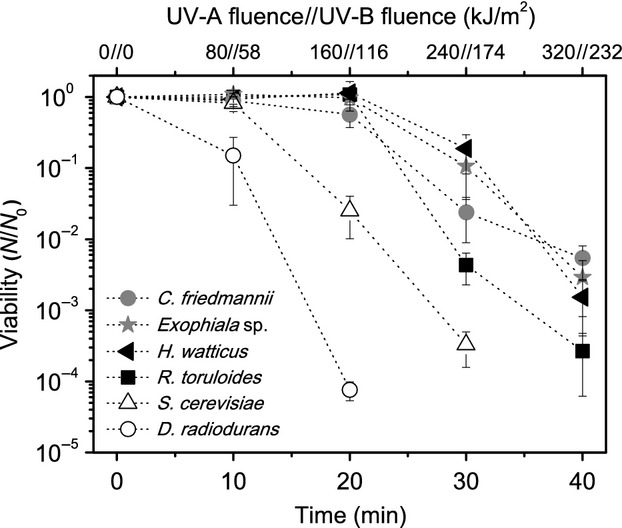
Survival curves for environmental-UV radiation of the tested organisms. The error bars indicate the variance between the triplicates.

All the isolated yeasts have presented significant resistance for simulated environmental-UV (Fig.5). Concerning the presence of melanin or carotenoids, the pigmented ones have not presented the best survival rates, suggesting that, at least for the tested species, other protection or repair mechanisms could be more important for environmental-UV resistance than pigmentation.

The presence of other photoprotective molecules like mycosporine (Libkind et al. 2009) or the expression of antioxidant enzymes that can efficiently scavenge ROS, may also be important in preventing the cellular malfunction generated by UV radiation (Hoerter et al. 2005). In addition, photoreactivation mechanisms might contribute to the repair of photoreaction damages to DNA, generated by UV light and constitute an important adaptation to endure such radiations (Zenoff et al. 2006).

Photoreactivation is generally performed by photolyases, enzymes that can repair CPDs and 6,4-PPs generated by UV-B and UV-C light. The repair can be induced by photosynthetically active radiation (PAR, 400–700 nm) or UV-A radiation and it has been demonstrated to be an important adaptive mechanism in high-altitude microorganisms (Zenoff et al. 2006; Albarracín et al. 2012). It is also known that Dewar lesions, generated under Solar irradiation, can be repaired by (6,4) DNA photolyases, although not very efficiently (Glas et al. 2010). In fact, all of the isolated yeasts have shown photorepair activity under the tested conditions in a qualitative experiment (Fig. S2). Using bioinformatics’ approaches, Lucas-Lledó and Lynch (2009) have already demonstrated that some fungi species seems to completely lack photolyase genes (*C. neoformans* and *Candida albicans*, e.g.) and argued that the low efficiency of natural selection on eukaryotic photolyases might be due to environments with low UV-incidence. Thus, the presence of such repair mechanism in our isolated yeasts can represent an adaptive advantage at the UV-rich Sairecabur environment. In addition, the capability of greater UV-C endurance via photolyases is interesting from an astrobiological point of view.

When exposed to environmental radiation, even the nonpigmented *S. cerevisiae* presented a better survival than the pigmented and radioresistant bacteria *D. radiodurans*. Indeed, it is known that *D. radiodurans*, even possessing carotenoids, is sensitive to UV-A radiation, mainly because of the singlet oxygen species generated by this kind of radiation (Slade and Radman 2011). This result suggests that even an organism that presents resistance to UV-B irradiation when tested with a lamp emitting spectral lines, may not necessarily be adapted to environmental-UV radiation. To ensure that this behavior was not caused due our experimental procedure, different growth phases of *D. radiodurans* were tested in saline solution, showing that is was consistently more sensitive than *S. cerevisiae* to environmental-UV in all cases (Fig. S4).

It should be noted that the differences in the biological response for the same fluence with the UV-B lamps and Solar simulator (as can be observed on the experiment depicted on Figs.3, 5) might reflect different underlying processes. As already pointed, the UV lamps produce intense spectral lines, in contrast to the broad continuous emission of the Solar simulator (Fig. S3). This can induce nonequivalent biological responses when both sources are compared, even for the same measured fluences, as the UV-B radiometer sensor cells used in this work, and on many others, have a range of sensitivity around 312 nm (280–320 nm). The UV-B lamps used have a peak emission at 312 nm, so most of the measured intensity correspond to this wavelength. The UV spectrum provided by the Solar simulator ranges from 280 nm to 400 nm, with a large proportion at the longer wavelengths, which have lower biological effectiveness (Horneck et al. 2006).

This explains why the UV-B radiation from the lamp was more effective than the UV-B radiation provided by the Solar simulator in inactivating the microorganisms. In any case, the direct comparison of the response to a line source, as the low-pressure Hg lamps for UV-B, to a broadband source, as the Solar simulator or natural Solar light, should be made with caution, as it has been already demonstrated that there are significant differences in microbial inactivation when comparing irradiated cells with polychromatic and monochromatic UV light (Zimmer and Slawson 2002). The Solar simulator represents better (and thus was used to mimic) the environmental conditions of Solar illumination on the surface of Earth, avoiding some of the bias of low-pressure lamps.

Melanized fungi isolated from the Antarctica samples have already been submitted to space and Martian-simulated conditions, presenting great resistance to such conditions (Onofri et al. 2008). Recently, a work by Zakharova et al. (2014) showed that black microcolonial fungi, including an *Exophiala* strain, were capable of maintaining metabolic activity under a simulated Martian environment, which shows the capability of eukaryotic microorganisms to be also good models for astrobiology, together with the prokaryotes.

## Conclusions

The capability of enduring high fluences of UV radiation is important when considering the potential for life as we know thriving on exposed surfaces of planetary bodies (Onofri et al. 2008; Abrevaya et al. 2011), such as Mars, icy moons or asteroids. In this work, we have isolated and characterized yeasts from a high-altitude area of the Atacama Desert, which are as resistant to UV radiation as the model organism *D. radiodurans*, one of the best candidates to survive in extraterrestrial conditions (Diaz and Schulze-Makuch 2006; Paulino-Lima et al. 2010). It was shown that the presence of melanin and carotenoids, as measured by Raman spectroscopy, does not always correlate with UV-resistance for the tested yeasts, especially for the case of environmental-UV, which is an important finding when considering the search for biosignatures on planetary surfaces, demanding additional studies. As the Atacama Desert is commonly considered a good Mars analog and the isolated extremophilic yeasts have demonstrated significant radiation and cold tolerance (growth at 0°C for three isolates and down to −6.5°C for two), these organisms can be used as model microorganisms to better understand the response of eukaryotes to extreme conditions, similarly to what has been proposed for the eukaryotes isolated from the Antarctica (Onofri et al. 2007). In this way, they can complement the existing models considered for astrobiological studies, and are already being applied in new multiparametric simulations of planetary surfaces and biosignature detection projects, with dedicated systems for this purpose (Rodrigues et al. 2012). In addition, although the objective of this work was not to characterize the full diversity of yeasts at the volcano, the species found at the samples are evidence of the potential of such sites for studying the diversity and global dispersion of cold-adapted yeasts.

## Supporting Information

**Figure S1.** (A) Growth curves of (◄) *Holtermanniella watticus*, (*) *Exophiala* sp. (●) *Cryptococcus friedmannii* at low temperatures with different solute concentrations. (B) Control growth curves performed at 15°C in: (■) TGY, (●) TGY + 0.5 mol/L NaCl, (▴) TGY + 0.4 mol/L glycerol, (▾) TGY + 0.6 mol/L glycerol.

**Figure S2.** Survival rates for all the isolates in different environmental conditions: (dark) Incubated in dark after UV-C irradiation; (PAR) incubated with photossinthetically active radiation after UV-C irradiation; (UV-A) exposed to UV-A radiation after UV-C irradiation. Methodology: Cells were grown and washed using the same protocol established for the UV-C and UV-B experiments. Several dilutions of the cells were then plated on TGY agar plates, which were pre-incubated for 45 min at 13°C for *Exophiala* sp. 15Lv1, *Holtermanniella watticus* and *Cryptococcus friedmannii,* and at 30°C for *Rhodosporidium toruloides*, ensuring they were at optimum temperature during the procedures. After the pre-incubation period, the plates were exposed to a single fluence of UV-C radiation, enough to diminish ∼90–99% of CFU/mL count (600 J/m^2^ for *H. watticus* and for *C. friedmannii,* 700 J/m^2^ for *Exophiala* sp. 15Lv1 and *R. toruloides*) under a flux of 14.5 W/m^2^ and then subjected to three different treatments: (1) Photoreactivation with PAR (400–700 nm); (2) UV-A photoreactivation (320–400 nm); (3) Dark incubation. For the dark repair treatment, the plates were immediately incubated in the dark after UV-C exposure. For the PAR photoreactivation treatment, after UV-C exposure, the plates were immediately transferred to a photoperiod incubator equipped with three fluorescent lamps (Osram 765 15W, Fig. S3 for the spectrum) which remained on during the whole incubation period, until the colonies were grown. For the UV-A photorepair treatment, after UV-C irradiation, cells were exposed to UV-A continuous radiation generated by an Oriel® Sol UV-2 Solar simulator equipped with an Oriel® SOL-UV-A-F filter to cut the UV-B portion of the spectrum (Fig. S3 for the spectrum), under the flux of 2.5 W/m^2^ (measured with the UV-A photocell) for 45 min. During this period, *R. toruloides* plates were kept at ∼30°C using a heating plate. For the other yeasts, plates were kept cold, at ∼13°C–10°C using a cold bath. The temperature of the plates during the irradiations was monitored using an electronic digital thermometer (HI-955502 Pt100, Hanna Instruments). After the UV-A exposure, plates were immediately incubated in the dark. We performed the irradiation of the cells directly on agar plates, since using liquid suspension implies in the post-manipulation of the cell suspension (for diluting it and platting), which might incur on light exposure, interfering on the photorepair assay. Experiments were performed in triplicates for each organism.

**Figure S3.** Spectra produced by the sources of radiation, as recorded using an Ocean Optics® QE65000 UV-Vis fiber-optic coupled spectrometer. (A) UV-C lamps, (B) Set of UV-B lamps, (C) Oriel® Sol UV-2 Solar simulator, (D) Oriel® Sol UV-2 Solar simulator equipped with Oriel® SOL-UV-A-F filter, (E) fluorescent lamps used on photoincubator. Notice the difference between the spectra (B and C) and the intense peak at 312 nm generated by the UV-B lamps, in contrast to a broad emission of the Solar simulator, more similar to the environmental UV radiation. We also show, in shaded light gray, the Vilber Lourmat UV-B photocell efficiency range. This illustrates that even with the same reading of flux at the radiometer, the UV-B spectra can differ significantly from each UV source, thus producing different biological responses.

**Figure S4.** Survival curves of *Deinococcus radiodurans* and *Saccharomyces cerevisiae* at different growth phases. The irradiation procedures were performed by growing the cells to the indicated OD_595_ (∼0.1, ∼0.7 and ∼2.3), washing the cells twice in 0.9% w/v NaCl solution and exposing the cell suspension to Solar irradiation, using the Oriel® Sol UV-2 Solar simulator. For the exposure, 600 *μ*L of the cell suspension were added to a 24-well plate and gently shaken during the irradiation procedure. The experiments were repeated twice, at different days, in duplicates for each experiment and yielded similar results. The survival was measured using the same methodology used on the UV-C, UV-B, and environmental-UV experiments. The data presented here is one of the two different repetitions and the error bars indicate the variance between duplicates.
