# Supplementary material for: UV-resistant yeasts isolated from a high-altitude volcanic area on the Atacama Desert as eukaryotic models for astrobiology
Source: Microbiologyopen. 2015 Jul 4;4(4):574–88. doi: 10.1002/mbo3.262 (PMC4554453; doi:10.1002/mbo3.262)
Supplement: Supplementary file 1 [file mbo30004-0574-sd1.pdf]

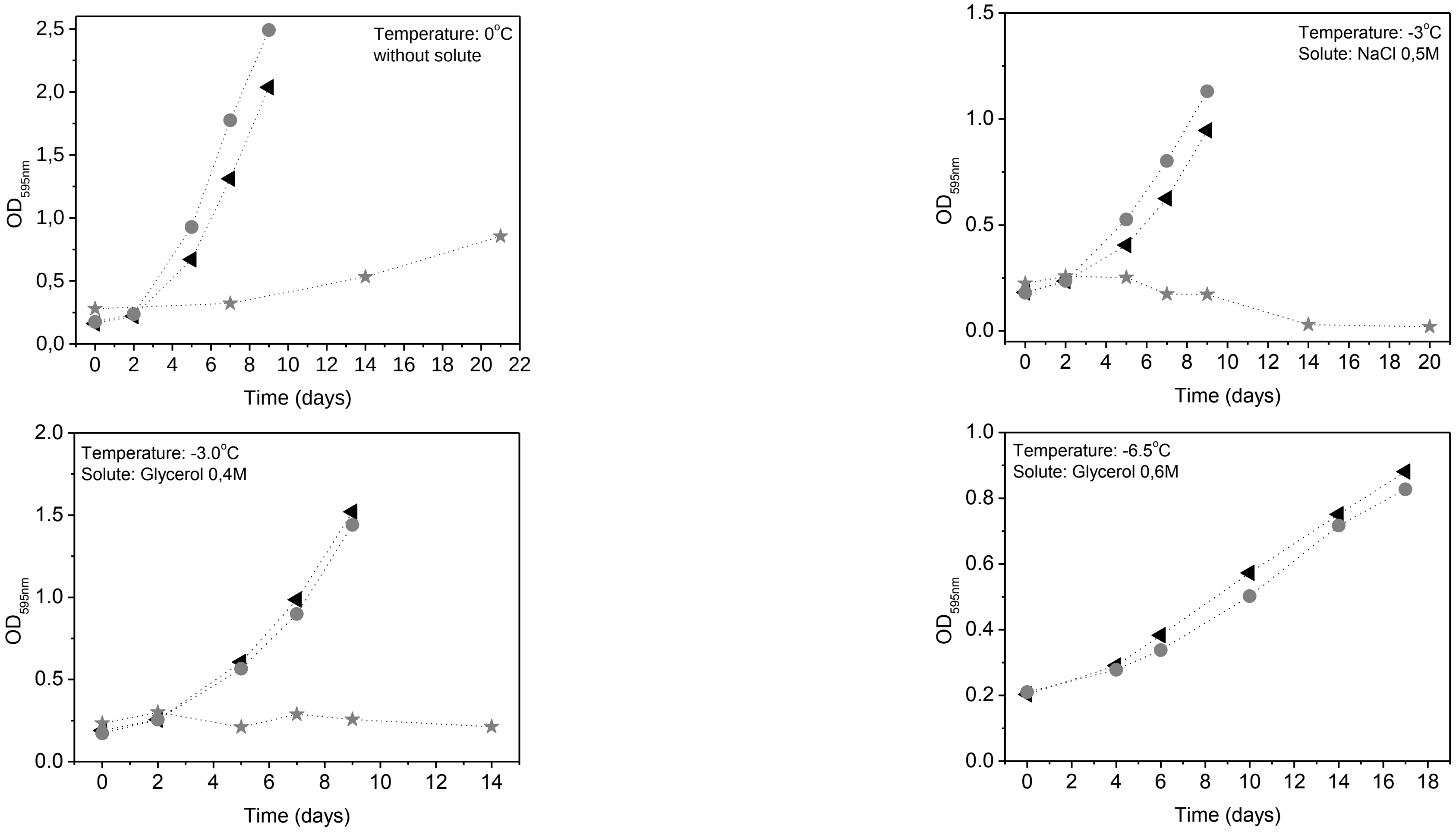

Supporting Figure 1A: Growth curves of (◀) *H. watticus*, (★) *Exophiala* sp. (●) *C.friedmannii* at low temperatures with different solute concentrations

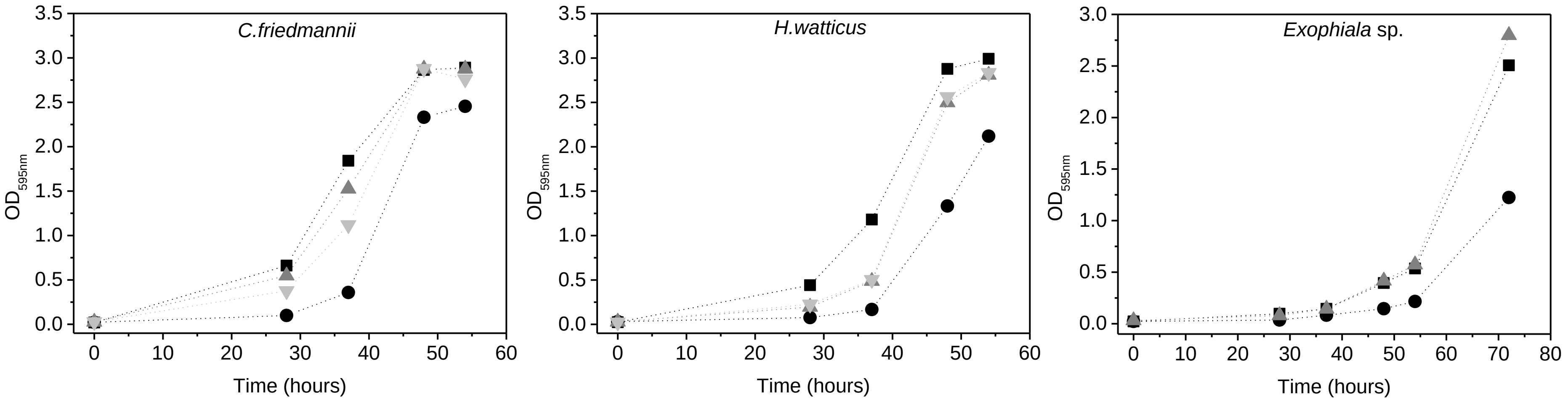

Supporting Figure 1B: Control growth curves performed at 15°C in: (■) TGY, (●) TGY + 0.5M NaCl, (▲) TGY + 0.4M glycerol, (▼) TGY + 0.6M glycerol.
