# Supplementary material for: UV-resistant yeasts isolated from a high-altitude volcanic area on the Atacama Desert as eukaryotic models for astrobiology
Source: Microbiologyopen. 2015 Jul 4;4(4):574–88. doi: 10.1002/mbo3.262 (PMC4554453; doi:10.1002/mbo3.262)
Supplement: Supplementary file 2 [file mbo30004-0574-sd2.pdf]

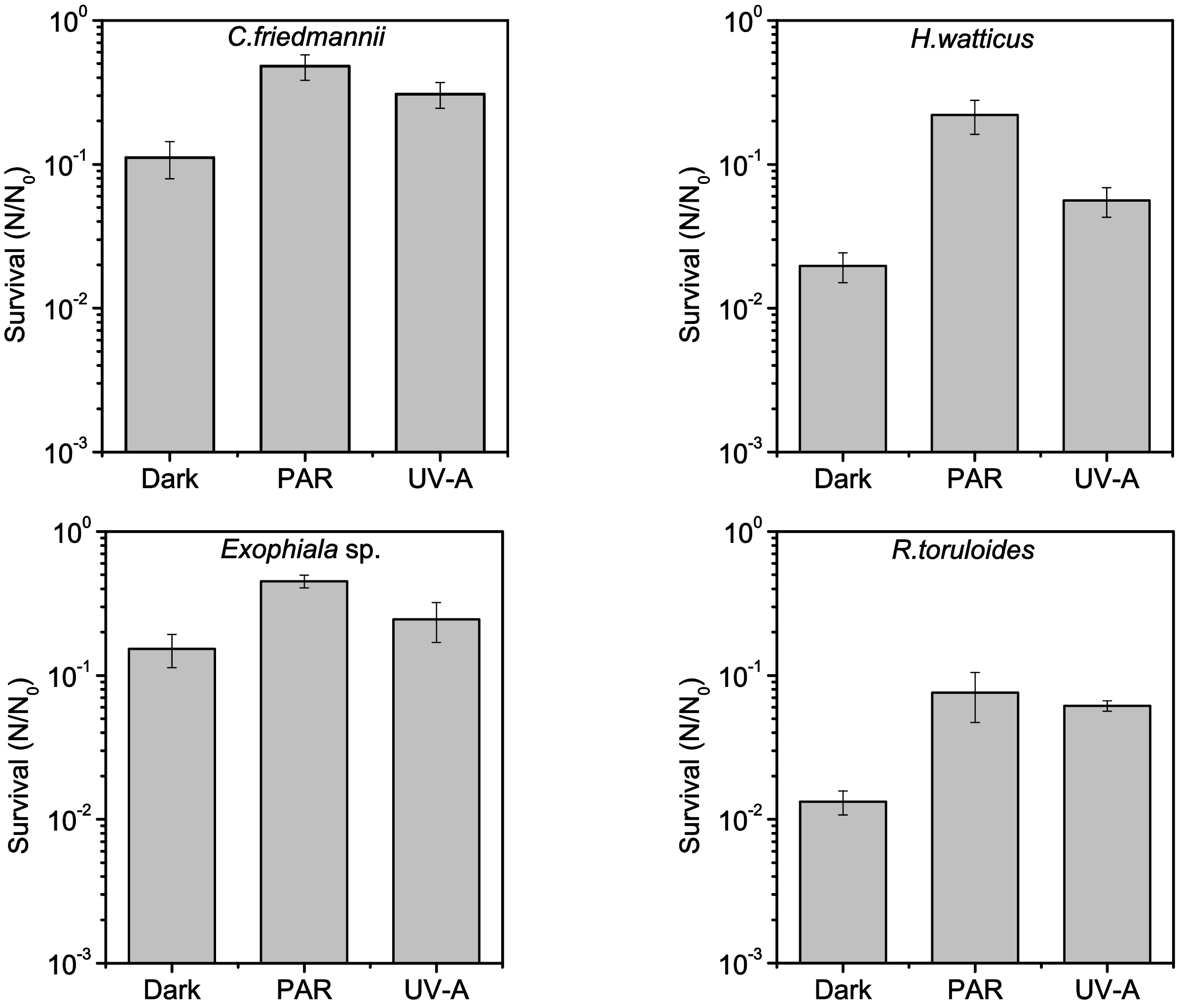

Supporting Figure 2: Survival rates for all the isolates in different environmental conditions: (dark) Incubated in dark after UV-C irradiation; (par) incubated with photossinthetically active radiation after UV-C irradiation ; (UV-A) exposed to UV-A radiation after UV-C irradiation.

Methodology: Cells were grown and washed using the same protocol established for the UV-C and UV-B experiments. Several dilutions of the cells were then plated on TGY agar plates, which were pre-incubated for 45 min at 13°C for *Exophiala* sp. 15Lv1, *H.watticus* and *C.friedmannii,* and at 30°C for *R. toruloides*, ensuring they were at optimum temperature during the procedures. After the pre-incubation period, the plates were exposed to a single fluence of UV-C radiation, enough to diminish ~90-99% of CFU/mL count (600J/m^2 for *H.watticus* and for *C.friedmannii* and 700J/m^2 for *Exophiala* sp. 15Lv1 and *R.toruloides*) under a flux of 14.5 W/m^2 and then subjected to three different treatments: (I) Photoreactivation with photosynthetically active radiation (PAR, 400-700nm); (II) UV-A photoreactivation (320nm-400nm); (III) Dark incubation. For the dark repair treatment, the plates were immediately incubated in the dark after UV-C exposure. For the PAR photoreactivation treatment, after UV-C exposure, the plates were immediately transferred to a photoperiod incubator equipped with 3 fluorescent lamps (Osram 765 15W, Supp. Figure 3 for the spectrum) which remained on during the whole incubation period, until the colonies were grown. For the UV-A photorepair treatment, after UV-C irradiation, cells were exposed to UV-A continuous radiation generated by an Oriel®Sol UV-2 Solar simulator equipped with an Oriel® SOL-UV-A-F filter to cut the UV-B portion of the spectrum (Supp. Figure 3 for the spectrum), under the flux of 2.5 W/m^2 (measured with the UV-A photocell) for 45 minutes. During this period, *R.toruloides* plates were kept at ~30°C using a heating plate. For the other yeasts, plates were kept cold, at ~13°C-10°C using a cold bath. The temperature of the plates during the irradiations was monitored using an electronic digital thermometer (HI-955502 Pt100, Hanna Instruments). After the UV-A exposure, plates were immediately incubated in the dark. We performed the irradiation of the cells directly on agar plates, since using liquid suspension implies in the post-manipulation of the cell suspension (for diluting it and platting), which might incur on light exposure, interfering on the photorepair assay. Experiments were performed in triplicates for each organism.
