# Supplementary material for: UV-resistant yeasts isolated from a high-altitude volcanic area on the Atacama Desert as eukaryotic models for astrobiology
Source: Microbiologyopen. 2015 Jul 4;4(4):574–88. doi: 10.1002/mbo3.262 (PMC4554453; doi:10.1002/mbo3.262)
Supplement: Supplementary file 3 [file mbo30004-0574-sd3.pdf]

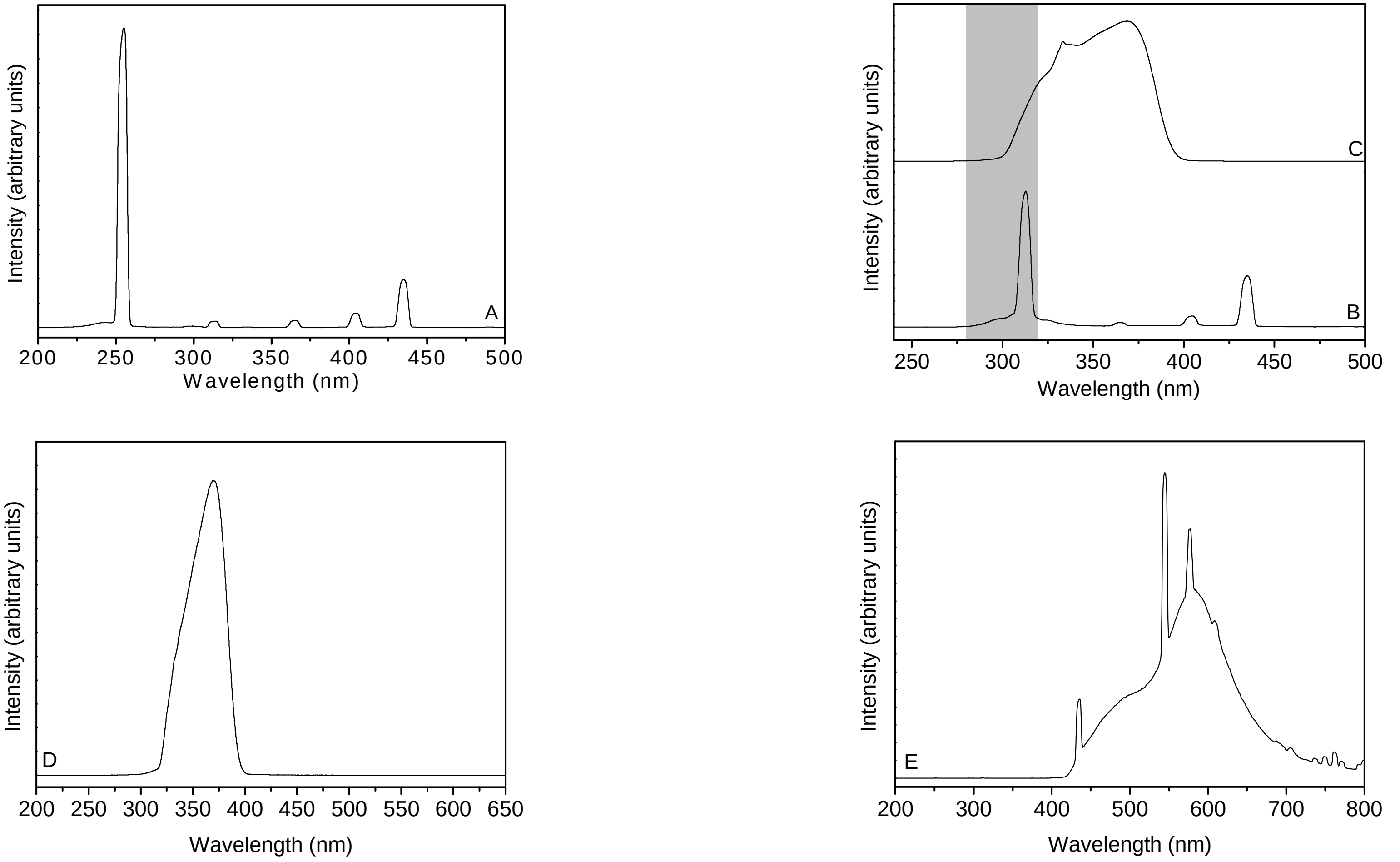

Supporting Figure 3: Spectra produced by the sources of radiation, as recorded using an Ocean Optics® QE65000 UV-Vis fiber-optic coupled spectrometer. (A) UV-C lamps, (B) Set of UV-B lamps, (C) Oriel® Sol UV-2 Solar simulator, (D) Oriel® Sol UV-2 Solar simulator equipped with Oriel®SOL-UV-A-F filter, (E) Fluorescent lamps used on photoincubator. Notice the difference between the spectra B and C, and the intense peak at 312 nm generated by the UV-B lamps, in contrast to a broad emission of the Solar simulator, more similar to the environmental UV radiation. We also show, in shaded light gray, the VilberLourmat UV-B photocell efficiency range. This illustrates that even with the same reading of flux at the radiometer, the UV-B spectra can differ significantly from each UV source, thus producing different biological responses.
