# Supplementary material for: UV-resistant yeasts isolated from a high-altitude volcanic area on the Atacama Desert as eukaryotic models for astrobiology
Source: Microbiologyopen. 2015 Jul 4;4(4):574–88. doi: 10.1002/mbo3.262 (PMC4554453; doi:10.1002/mbo3.262)
Supplement: Supplementary file 4 [file mbo30004-0574-sd4.pdf]

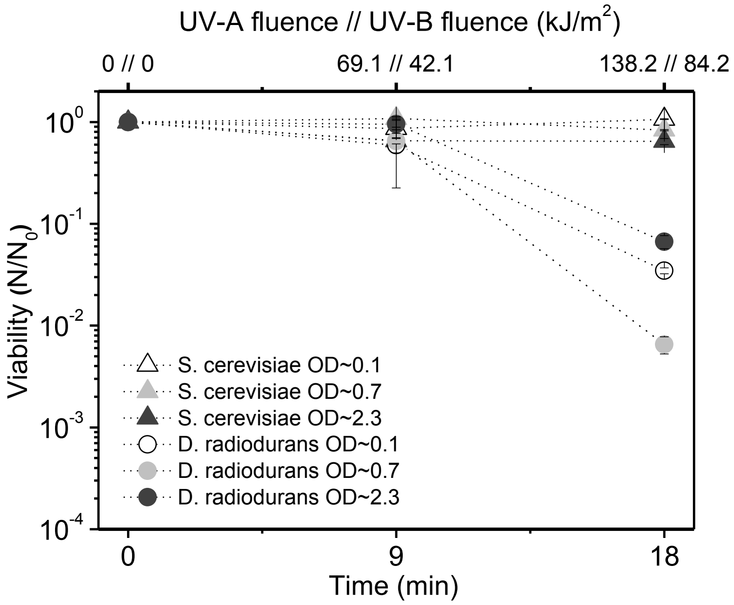

Supporting Figure 4: Survival curves of *Deinococcus radiodurans* and *Saccharomyces cerevisiae* at different growth phases. The irradiation procedures were performed by growing the cells to the indicated OD_{595} (~0.1, ~0.7 and ~2.3), washing the cells twice in 0.9% w/v NaCl solution and exposing the cell suspension to Solar irradiation, using the Oriel® Sol UV-2 Solar simulator. For the exposure, 600 μl of the cell suspension were added to a 24-well plate and gently shaken during the irradiation procedure. The experiments were repeated twice, at different days, in duplicates for each experiment and yielded similar results. The survival was measured using the same methodology used on the UV-C, UV-B and environmental-UV experiments. The data presented here is one of the two different repetitions and the error bars indicate the variance between duplicates.
